# A web-based protein interaction network visualizer

**DOI:** 10.1186/1471-2105-15-129

**Published:** 2014-05-06

**Authors:** Gustavo A Salazar, Ayton Meintjes, Gaston K Mazandu, Holifidy A Rapanoël, Richard O Akinola, Nicola J Mulder

**Affiliations:** 1 Computational Biology Group, IDM, Faculty of Health Sciences, University of Cape Town, Anzio Road, Cape Town, South Africa

**Keywords:** Visualization, Protein-Protein Interactions, PPI, Web development

## Abstract

**Background:**

Interaction between proteins is one of the most important mechanisms in the execution of cellular functions. The study of these interactions has provided insight into the functioning of an organism’s processes. As of October 2013, *Homo sapiens* had over 170000 Protein-Protein interactions (PPI) registered in the Interologous Interaction Database, which is only one of the many public resources where protein interactions can be accessed. These numbers exemplify the volume of data that research on the topic has generated. Visualization of large data sets is a well known strategy to make sense of information, and protein interaction data is no exception. There are several tools that allow the exploration of this data, providing different methods to visualize protein network interactions. However, there is still no native web tool that allows this data to be explored interactively online.

**Results:**

Given the advances that web technologies have made recently it is time to bring these interactive views to the web to provide an easily accessible forum to visualize PPI. We have created a Web-based Protein Interaction Network Visualizer: PINV, an open source, native web application that facilitates the visualization of protein interactions (http://biosual.cbio.uct.ac.za/pinv.html). We developed PINV as a set of components that follow the protocol defined in BioJS and use the D3 library to create the graphic layouts. We demonstrate the use of PINV with multi-organism interaction networks for a predicted target from *Mycobacterium tuberculosis*, its interacting partners and its orthologs.

**Conclusions:**

The resultant tool provides an attractive view of complex, fully interactive networks with components that allow the querying, filtering and manipulation of the visible subset. Moreover, as a web resource, PINV simplifies sharing and publishing, activities which are vital in today’s research collaborative environments. The source code is freely available for download at https://github.com/4ndr01d3/biosual.

## Background

Multiple experiments can be used to predict an interaction between proteins, and the outcome of these have been continuously deposited in public resources such as IntAct [1]. Often a single protein can have dozens of reported interactions, which implies a rapid escalation of the amount of data for analysis involving several proteins (e.g. pathways). A common strategy for making of this data is to generate graphic representations of the interaction networks, where some features become evident at a glance, for instance identification of the nodes that have the highest number of interactions or finding a common protein that interacts with the rest.

There are several software tools that allow the visualization of protein interactions, including Cytoscape [2,3], Networkx [4] and NAVIGaTOR [5,6]. Each of these tools provide different methods to visualize protein networks, and their strengths and weaknesses have been discussed in several earlier reviews [7-10]. Nonetheless, we consider that none of the current options provides the convenience or benefits available in a native web application.

Until recently it would not have been realistic to conceive of a web application that could cope with the requirements imposed by a visualization tool for protein interactions. However, due to recent developments (such as the set of technologies grouped under the term HTML5), this is now possible: the data can be acquired using a request via AJAX; it can be processed and injected into the current document with JavaScript; the document can contain structured graphics in SVG format; the styles defined with CSS3; and all within reasonable response time thanks to the most recent generation of browsers.

We took advantage of the above mentioned features to develop PINV: Protein Interaction Network Visualizer, an HTML5 application that runs on every modern browser, whose function is to allow the visualization and exploration of proteins and their interactions. Researchers using PINV can access their visualizations from any computer with an internet connection and a modern browser without the need for installation of any third party software or plugins; moreover the nature of the Web simplifies tasks such as sharing and publishing visualizations.

## Implementation

The PINV architecture is based on the composition of BioJS components. BioJS is a community project to create a library of visual JavaScript components for biological sciences [11]. The main layout components of PINV are available in the BioJS registry [12], and were developed using D3 (Data-Driven Documents) [13], a JavaScript library for the manipulation of data and its visualization through web components.

PINV uses a Solr search engine server [14] in order to perform rapid queries, resulting in quicker response times than through querying of a relational database. This requires that the interaction dataset is pre-loaded onto our servers. Currently we support web submission of data as a pair of files: one for interactions and one for features, both of which are simple tab separated files.

In the interaction file, the first two columns contain the accession numbers of the proteins, and the third column is an aggregate score of the evidence for the reported interaction. Subsequent columns are optional and considered as evidence scores that contributed to the unified value in column 3. The second file is used to add annotations to the proteins (e.g., gene name). The only required annotation is that of the organism to which the protein belongs; therefore the file should have the protein id in the first column, the organism in the second and any other features in additional columns. The information provided in this file can then be used to manipulate the graphic, for instance to color by functional class or to show a particular label. The support of other file types is part of the development roadmap along with the ability to recover the interactions from public databases, and the annotations directly from UniProt.

When uploading data, the user can choose between two modes of privacy: public and protected. A dataset in public mode will be listed on PINV’s website and accessible to anyone. This brings visibility to the data and promotes collaboration between researchers. On the other hand, protected mode ensures that only users with a valid link can retrieve information from the data set; the link includes a unique key that is sent to the uploader so he can distribute the link as he wishes. In this way the collaborative features of PINV are still functional between the holders of the link.

We have loaded some public datasets for organisms of our particular interest: *Homo sapiens* (HS), *Mycobacterium leprae* strain TN (MLP), *Mycobacterium smegmatis* strain MC ^2^155, *Mycobacterium tuberculosis* strain CDC1551 (MTB) and a combined dataset of *HS*, *MTB* and the interactions between them.

## Results

**Figure 1:**
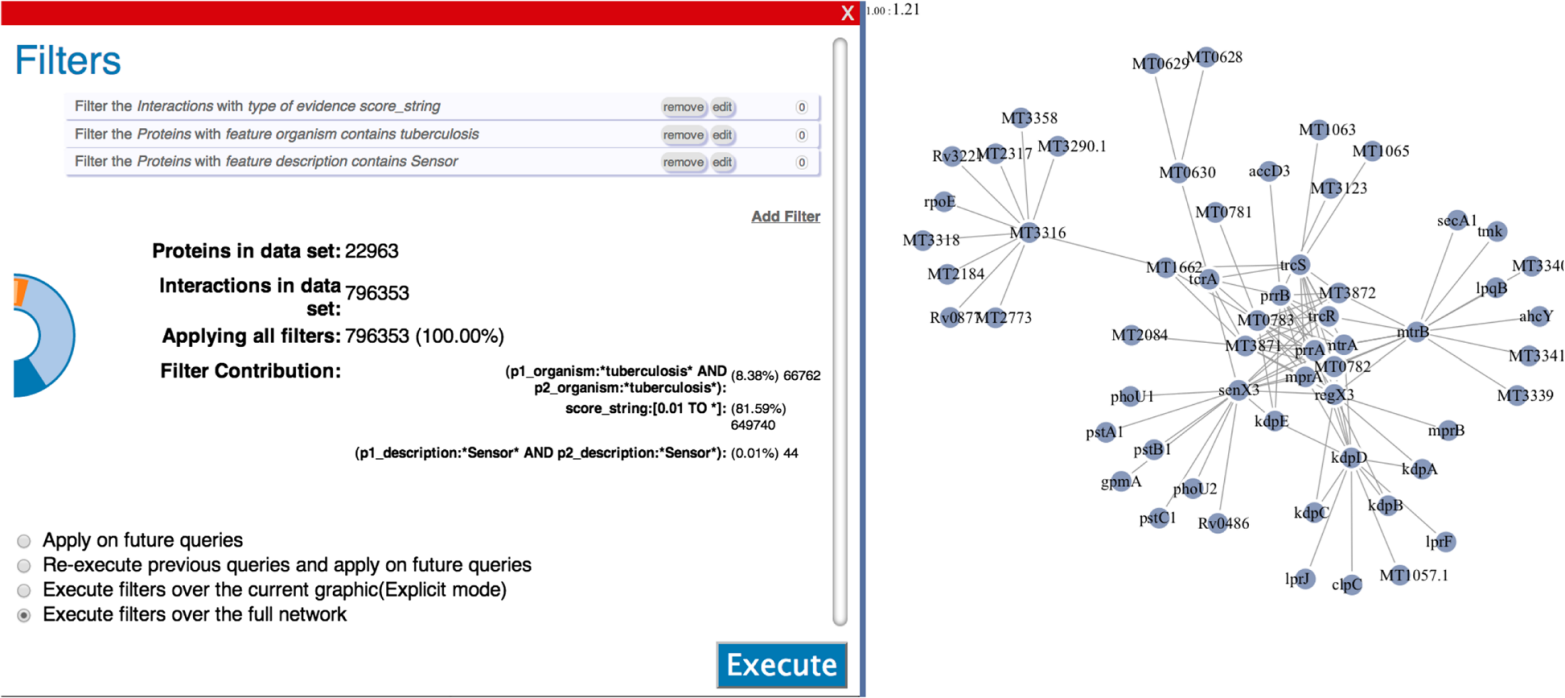
**PINV Filters Dialog.** Snapshot of the dialog where the user can explore a dataset by defining filters.

In order to meaningfully visualize protein interactions, it is beneficial to reduce the network to only those proteins of interest through the application of prefilters to the data. This has the simultaneous benefits of increasing the speed of PINV’s response time and reducing bandwidth requirements. By using the prefilter tool on PINV, the user can explore the whole content of the network without loading the individual components into the graphic. Figure 1 (Left) shows the interface where the user can define certain conditions that the interactions have to fulfil in order to be displayed. The graphic on the left side of Figure 1 is dynamically updated, reflecting the size of the subset generated by applying the filters over the whole dataset. For instance, in the graphic the dark blue represents the whole network (796353 interactions), while the light blue is the portion of those that have an evidence score from STRING [15]. Two more filters were applied to this example (*organism contains tuberculosis* and *description contains Sensor*), the combined use of the filters results in a 103 interaction subnetwork, which is small enough to allow PINV to run smoothly and generates a graphic like that in Figure 1 (Right).

**Figure 2:**
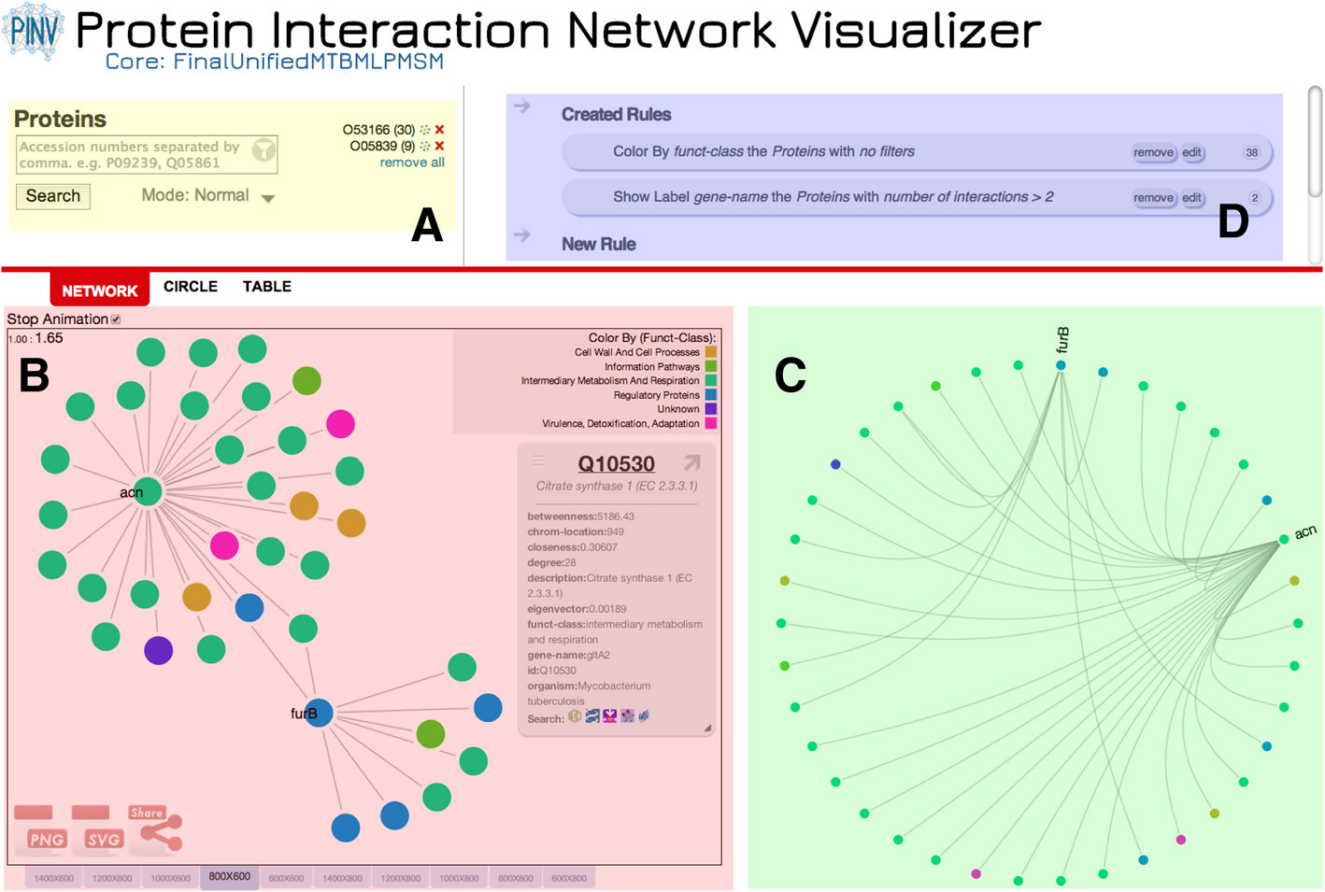
**PINV Snapshot.(A)** Search Form. **(B)** Network layout. **(C)** Circle layout **(D)** Manipulation by Rules.

The prefilters tool is the first window a user sees once a dataset has been selected on PINV’s homepage. The user can choose not to use any prefilters and close the window, which will display the main graphical interface of PINV (Figure 2).

As an alternative to the prefilters, the application also provides a search form where the proteins of interest can be selected by inserting comma separated accession numbers. The form has auto-complete capabilities to assist in this process (Figure 2-A). The icon at the top right of this area allows users to open the prefilters window at any time. The search function has three modes: Normal, Explicit and Recursive. The *Normal* mode returns the queried proteins and all interactions reported in the dataset, while the *Explicit* mode will only display proteins that have been explicitly included in the query list and the interactions between them; the explicit mode will also include all the interactions with the previous results. The *Recursive* mode combines the previous two, it first gets the target proteins on *Normal* mode, and then for each recovered protein it triggers a query in *Explicit* mode, the outcome of this is that all the interactions between displayed proteins will be shown.

PINV provides three types of visualization of the data: Network, Circle and Table. Figure 2-B displays a snapshot of the interactions for proteins O53166 and O05839 from the MTB dataset in the Network layout.

The network layout allows the re-organization of the proteins through drag and drop mouse gestures, and clicking on a node will display the loaded features that are hyperlinked to relevant public resources in a pop-up window (right side of Figure 2-B). The proteins in this layout are self-organised through the D3 implementation of a force layout. This simulates a repulsion force between nodes without a link and an attraction force where there is a link. The outcome of this algorithm is a network that clusters the highly connected proteins and makes evident the connections between these groups. It is also possible to impose a center of gravity in the graphic, where nodes are attracted. We have used this feature to create as many gravity points as organisms are present in the current selection, resulting in the separation of proteins from different organisms.

The second layout organizes the proteins in a circle positioning all the nodes around the center without favouring any of them, this can help to discover connection patterns around the network (Figure 2-C). In this view the interactions of a single protein are highlighted when the user hovers over the node. As in the network layout, the proteins are separated to clearly indicate the organism to which they belong; this feature will be extended to allow the user to choose the parameters to group the proteins around the circle.

Lastly there is the table view where the raw data is displayed and can be reorganized. This is particularly useful for queries that return too many results and require post-filtering to select the proteins of interest.

The visualization of the layout can be exported at any time into SVG or PNG format, and the data in the table are exportable to a CSV file.

Manipulation of any of the three display options is achieved with a single component (Figure 2-D) that allows the creation of rules. The rationale behind this component is that by specifying a target (protein or interactions), a condition to filter by (e.g., protein with functional class X, interactions with protein Y), and an action (e.g., paint green, show label) it is possible to manipulate a wider set of network elements with less input from the user. For example, Figures 2-B and C show all the proteins painted in different colors depending on their functional class. The legend on the right lists the functional classes used and the corresponding colors in the graphic. A second rule was created in the example to display the gene name at the side of proteins with more than 2 interactions. Right clicking on a protein in the graphic will open a contextual menu that allows the quick creation of rules that only apply to the selected protein. Users can also color nodes with quantitative data such as expression values loaded from a third file.

All the actions executed by the user have been saved in order to display the provenance of the visualization in the form of a list where the user and collaborators can review the steps (and their parameters) taken to reach a particular status of the visualization. This list can be displayed by clicking on the “Show History” button at the bottom of the page. We plan to extend this to allow the user to jump to a particular state in the history.

PINV provides an easy way to share the current view: when the user clicks on ‘share’, a unique link is generated. PINV is able to regenerate the status of all the components to re-compose the view whenever the link is used. This functionality was developed with the goal of promoting collaboration between users. If for instance, a user finds something of interest in the visualization, he/she can create the link and send it to a collaborator via mail, instant messaging, etc. The second researcher can not only view but manipulate the visualization and re-create another link for future discussions. For example, recreations of Figures 2 and 1 can be explored in PINV by following the links [16,17] respectively.

The sharing option also provides an HTML snippet that can be used to embed the view in a web page, making it easier for the researcher to include their visualizations on web documents in the same way that maps, videos and other kinds of multimedia are included in blog posts. It is hoped that researchers will use this to embed PINV graphics in pages that discuss the results of their own network research.

## Discussion

### Selection of the technology to generate the graphics

Currently, the dynamic creation of graphics according to web standards can be generated using two different techniques: Scalable Vector Graphics (SVG) and Canvas. Both are well supported in modern browsers and their manipulation is possible with standard JavaScript.

SVG is a markup language like HTML, and as such, each component of the graph is represented as an element of the document and is held in memory in the browser as part of the Document Object Model (DOM). Because of this, the whole content of the graphic can be embedded in the same document as its parent web page. Canvas, on the other hand, is a single element in the web document, whose content manipulation is carried out via JavaScript. Only the final result is stored in memory. As a consequence, Canvas requires less memory than SVG, but lacks the control per element that SVG offers [18].

We chose to use SVG in the development of PINV because of the atomic control over the elements: proteins (SVGPathElement) and interactions (SVGLineElement), which allows us to bind events and apply styles in the same way as any other HTML element.

There are JavaScript libraries for Canvas that allow manipulation of the elements painted on the Canvas, however the poorer quality of zooming and the lack of styling via CSS are factors that motivated us to choose SVG.

The result of not using Canvas is evident in PINV when there are a large number of elements to display (depending on the machine this can number around one thousand interactions). The fact that SVG holds the information of all the components in the DOM is memory intensive because it stores data for components that can be hidden, are out of focus or so small that they are not actually visible. In contrast, a Canvas strategy is able to deal with many more elements. For this reason we have included the study of the performance of using Canvas on PINV instead of SVG (or as a hybrid solution) in our road map for future development e.g. the use of Canvas when there are more than X elements, otherwise using SVG.

### Comparison with similar tools

Most interactive online visualization tools only allow the viewing of networks that are preloaded, meaning that the user cannot view a network he/she has generated.

The online version of Graphle [19] is limited to graphs generated by bioPIXIE in yeast, MEFIT in *E. coli*, or HEFalMp for human data. STRING [15], a database for known and predicted protein-protein interactions, and STITCH [20], its sister project for chemical-protein interactions, integrate interaction information from various sources in addition to in-house predictions. Both projects use the same library to display their data. However, the network visualized in both databases is barely customizable. The public implementation of VisANT [21], a web-based (via Java Applets) workbench for the integrative analysis of biological networks, is based on the Predictome database. PINV on the other hand, allows the user to view preloaded data but also to load their own data.

Another alternative is Cytoscape Web [22]. However, the fact that its introduction tutorial [23] requires its users to code in JavaScript indicates that this tool is mainly intended for developers to display networks on the web, not for the scientist who has a network to visualize. In this regard, Cytoscape Web was considered as an alternative to D3 for our background technology to generate the graphics as it is closer to the biological concepts tied to this tool. Unfortunately Cytoscape Web uses Adobe Flash for the generation of the graphics, which goes against our objective of providing a native web application (i.e. developed using recent web standards). A more recent development is Cytoscape.js [24]. The principles behind this project are similar to those followed by PINV: A modern web toolset to display interactions. Nonetheless, the two projects differ from one another as Cytoscape.js is a library for programmers, while PINV is an application. A combined strategy of Cytoscape Web and its well known stand alone parent project is discussed further below.

We also compared PINV with the stand-alone version of Cytoscape by using the network in the biological example mentioned in the following section. The original network contains all interactions from the three organisms and the orthologs with a total of 165,000 interactions. The performance of Cytoscape notably exceeded PINV when dealing with a network of this size. Moreover, there are considerably more manipulation options in Cytoscape than the ones currently provided by PINV. However, Cytoscape and its many plugins need to be installed by the user and any collaborator sharing the results.

**Figure 3:**
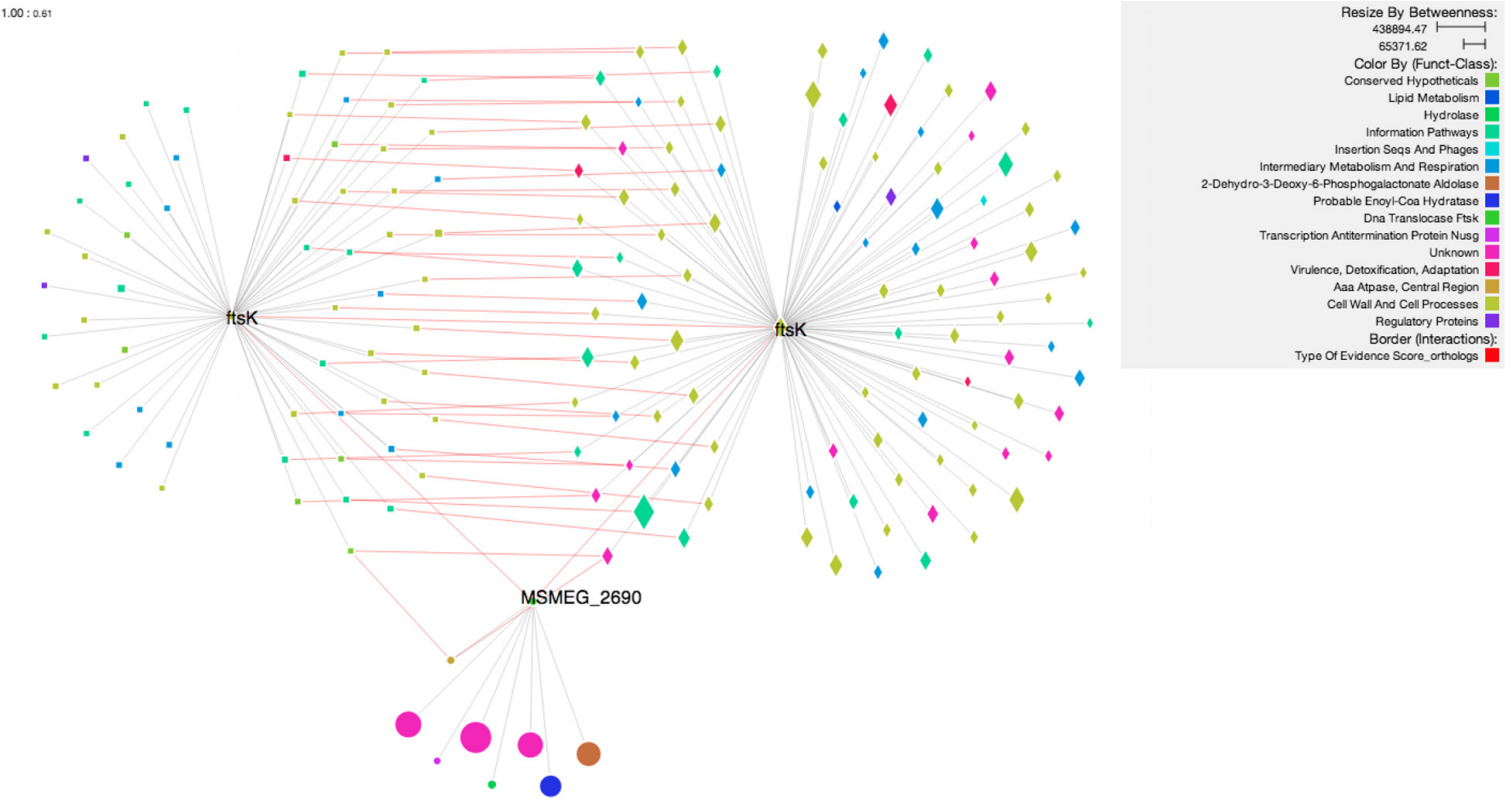
**Orthologs in 3 organisms.** The FtsK protein from *Mycobacterium tuberculosis* with its orthologs (Interactions in red) in *M. leprae* and *M. smegmatis*.

Cytoscape Web includes a *showcase* demo that allows files to be opened in several formats. In order to reproduce the same graphic as in Figure 3, we preprocessed the network on the stand-alone tool in order to filter it, and focus on the target orthologs. This step was necessary because Cytoscape web fails when loading a network of this size. Subsequently, we successfully uploaded the subnetwork. Filters and styles can be manipulated online but extending the subnetwork requires loading of additional data.

We are aware that PINV will also struggle to display the 165,000 interactions of the network. However, the strategy of only visualizing by request and the use of prefilters allows the user to navigate the network by limiting the graphic to the interactions of interest. In contrast Cytoscape web does not provide tools to explore a large dataset and requires the use of the stand-alone application (or other software) in order to filter and create a subnetwork.

### A biological use case scenario

To illustrate the richness of information that can be conveyed using PINV graphics, we use the biological example of *Mycobacterium tuberculosis* (MTB), its host *Homo sapiens* (HS), and the two related mycobacteria species *M. leprae* (MLP) and *M. smegmatis* (MSM). In particular, we look at the DNA translocase protein FtsK (UniProt accession O33290), which coordinates cell division and chromosome segregation in MTB, and is considered a high confidence drug target. We view this protein in the context of its interaction network within MTB, its host-pathogen interactions with proteins in HS, and orthologous proteins in MSM and MLP.

Figure 3[25] shows FtsK and its interactors in MTB, along with orthologous proteins (and their interactors) in MSM and MLP. Using custom rules to control the rendering of elements, we are able to highlight various annotations such as species (by node shape and placement), interaction type (by line color), functional class (by node color) and the network property of betweenness (by node size). The higher number of orthologs shared between MTB and MLP is immediately evident in the graphic, and reflects the closer relationship between these two species, which are both slow-growing bacteria in contrast to the fast-growing MSM.

**Figure 4:**
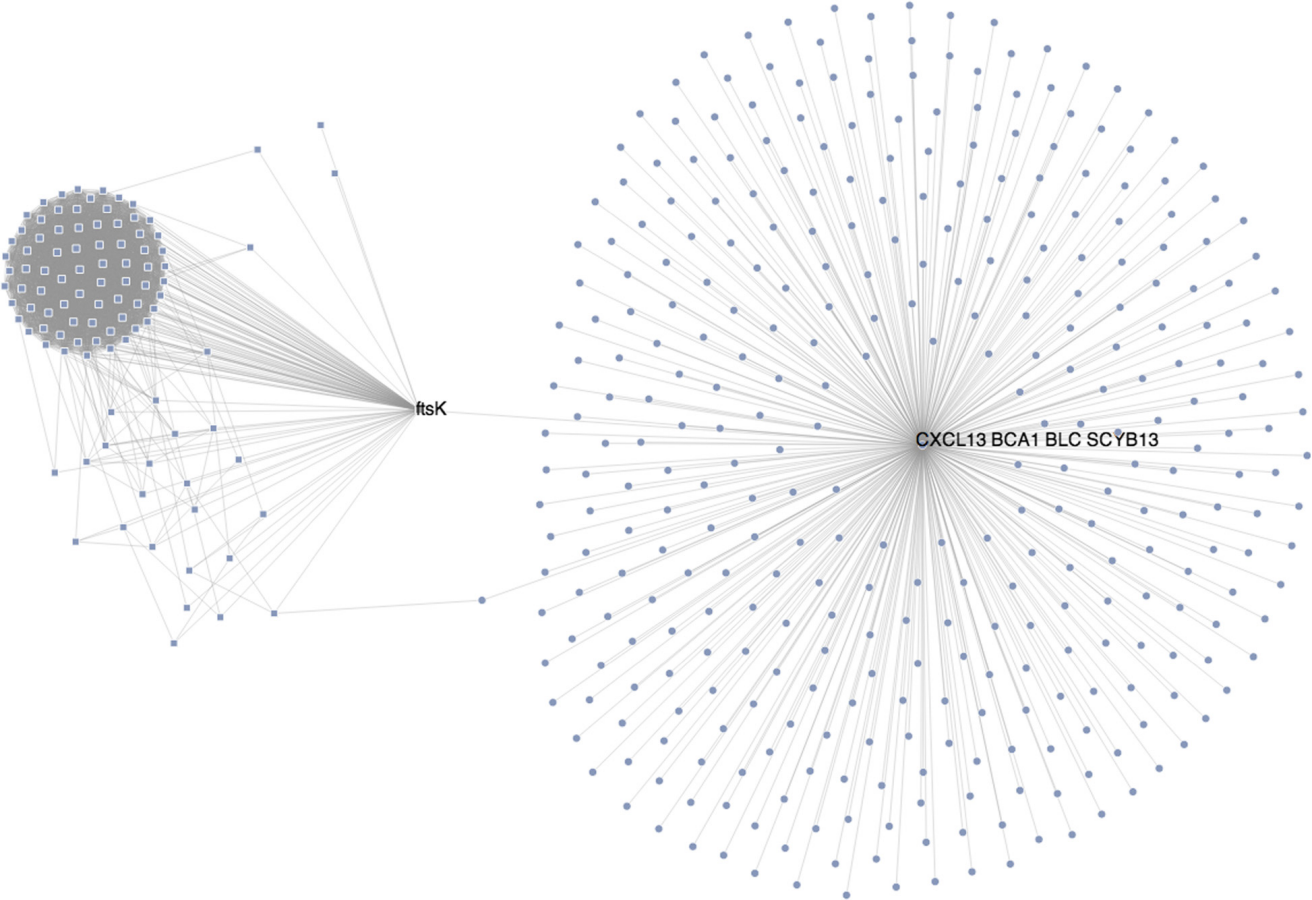
**Pathogen and host proteins.** The same protein FtsK interacting with a protein in *Homo sapiens* (the host organism).

Figure 4[26] shows FtsK with its MTB interactions again, this time in the context of host-pathogen interactions with *Homo sapiens* proteins. A recursive mode query has been used to extract and highlight multiple connections between subnetworks in MTB and HS, specifically FtsZ’s direct interaction with HS’s C-X-C motif chemokine 13, as well as an indirect path to the same HS protein via the MTB protein Q7D8P2 and its interaction with the human tyrosine aminotransferase.

Both of these visualisations were created using simple queries and display rules, and can easily be shared in their fully interactive form with a simple URL. An example of the former graphic, embedded in a third-party web page can be found here [27].

## Future work

The native web nature of PINV offers several opportunities for future enhancement. HTML5 is well supported by most smartphones and tablets. PINV can currently be displayed on these platforms, however requires modifications to the event handling to accomodate their touch interfaces. We think that the inclusion of mobile technologies will contribute to a more collaborative environment between researchers.

The current architecture of PINV uses Solr on the server side to effectively store and query the interactions. Additionally, there is logic on the server side to manage the uploading of datasets, the saving of graphic status for the sharing and controlling of access to protected datasets. We are using CherryPy as the middleware language to deal with these functionalities. This adds complexity to the process of installing PINV on a private machine, which might be desired by users who want total privacy of their data. An alternative that we are considering implementing is providing an option to use a client-side storage capability: IndexedDB. By using IndexedDB, users won’t be uploading their data to our servers, instead, all the information will be kept on the same machine where the user is opening the website. Once open, PINV will be able to run off-line as both software and data will be on the client machine. However this change requires a full restructure of the methods used to query the data sets because the queries in IndexedDB are built in a different way to those in Solr.

Another feature on the roadmap of PINV is the enrichment of the visualization with online resources that provide information about the proteins and/or interactions. For example, annotations can be added to each protein via online queries to sources such as UniProt, and additional interaction evidence can be retrieved from other PPI databases.

A major challenge in the visualization of PPI networks is the graphic representation of a large number of interactions without it collapsing into the infamous hair ball. Apart from the technological challenges of processing large volumes of data, the resulting graphic is often too complex to provide insights into the nature of the network. Besides the option to select a subnetwork of interest based on prefilters, we are investigating different techniques (e.g. clustering) to generate high level representations of big networks in a way that both improves performance and gives an overall idea of the structure of a network with thousands of proteins. This will facilitate easier visual mining of the data.

## Conclusions

PINV is an open source, native web application that uses the latest generation of web technologies to offer an interactive view of protein-protein interactions which is easily accessible from any modern browser. PINV enables researchers to explore their data using different methods and the visualization can be manipulated to highlight the proteins or interactions of interest. The resulting graphic can be exported to common graphic formats, shared via URL or embedded in third-party pages, features that make it suitable for publication, sharing and collaboration activities.

We consider PINV to be a unique tool for visualization and exploration of PPI networks, not only in terms of the technology used (HTML5) but also because today’s research requires collaborative efforts to process data. PINV offers an intuitive way to visualize, share and publish PPI data without the need to install any extra software (besides a web browser) and/or third party components such as Flash or Java.

## Availability and requirements

**Project name:** PINV: Protein Interaction NetworkVisualizer 

**Project home page:**http://biosual.cbio.uct.ac.za/pinv

**Source code:**https://github.com/4ndr01d3/biosual

**Operating system(s):** Web based, Platform independent. 

**Programming language:** HTML5: HTML + CSS +JavaScript 

**Other requirements:** Modern Browser (2012+) 

**License:** LGPL 

**Any restrictions to use by non-academics:** None

## Abbreviations

AJAX: Asynchronous JavaScript and XML; CSS: Cascading style sheets; CSV: Comma-separated values; D3: Data-driven documents; HTML: HyperText markup language; PINV: Protein interaction network visualizer; PNG: Portable network graphics; PPI: Protein-protein interaction; SVG: Scalable vector graphics; URL: Universal resource locator.

## Competing interests

The authors declare that they have no competing interests.

## Authors’ contributions

Critical revision of the manuscript for important intellectual input: GS, AM, GM, HR, RA and NM. Study concept: GS, AM and NM. Software Design: GS, AM, GM, HR, RA and NM. Software development: GS and AM. Creation of datasets: GM, HR and RA. Software Testing: GM, HR, RA and NM. Software Documentation: GS and RA. Drafting of the manuscript: GS, HR and NM. Supervision: NM. All authors read and approved the final manuscript;
